# Editor's Letter-Box

**Published:** 1899-11-25

**Authors:** 


### EDITOR'S LETTER-BOX.

Referring to an article in The Hospital for November 11th on "Injuries to the Spine," in which a paper by Mr. Noble Smith was alluded to, Mr. Noble Smith writes to say that the main object of that paper was not, as was represented, "to advocate the use of a particular appliance." He says: "Its object was to advocate the following principles of treatment: (1) Reduction of the displacement, when possible, in nearly all cases of fracture; (2) efficient fixation; (3) operative measures in every case in which the least prospect presents itself of thus affording relief of pressure upon the spinal cord, provided the symptoms do not denote destruction of that part. I described the splint which I have found most efficient for fixation, but I also referred to other methods of obtaining a similar result."

